# MicroRNA expression signature in human abdominal aortic aneurysms

**DOI:** 10.1186/1755-8794-5-25

**Published:** 2012-06-15

**Authors:** Matthew C Pahl, Kimberly Derr, Gabor Gäbel, Irene Hinterseher, James R Elmore, Charles M Schworer, Thomas C Peeler, David P Franklin, John L Gray, David J Carey, Gerard Tromp, Helena Kuivaniemi

**Affiliations:** 1 The Sigfried and Janet Weis Center for Research, Geisinger Clinic, 100 North Academy Avenue, Pennsylvania, 17822-2610, USA; 2 Department of Biology, Susquehanna University, Selinsgrove, PA, USA; 3 Department of Visceral, Thoracic and Vascular Surgery, Technical University of Dresden, Dresden, Germany; 4 Department of Vascular and Endovascular Surgery, Geisinger Clinic, Danville, PA, USA; 5 Department of General, Visceral, Vascular and Thoracic Surgery, Charité Universitätsmedizin, Charité Campus Mitte, Berlin, Germany

**Keywords:** Apoptosis, Microarray analysis, Vascular biology, miRNA-mRNA analysis, Network analysis

## Abstract

****Background**:**

Abdominal aortic aneurysm (AAA) is a dilatation of the aorta affecting most frequently elderly men. Histologically AAAs are characterized by inflammation, vascular smooth muscle cell apoptosis, and extracellular matrix degradation. The mechanisms of AAA formation, progression, and rupture are currently poorly understood. A previous mRNA expression study revealed a large number of differentially expressed genes between AAA and non-aneurysmal control aortas. MicroRNAs (miRNAs), small non-coding RNAs that are post-transcriptional regulators of gene expression, could provide a mechanism for the differential expression of genes in AAA.

****Methods**:**

To determine differences in miRNA levels between AAA (n = 5) and control (n = 5) infrarenal aortic tissues, a microarray study was carried out. Results were adjusted using Benjamini-Hochberg correction (adjusted *p* < 0.05). Real-time quantitative RT-PCR (qRT-PCR) assays with an independent set of 36 AAA and seven control tissues were used for validation. Potential gene targets were retrieved from miRNA target prediction databases Pictar, TargetScan, and MiRTarget2. Networks from the target gene set were generated and examined using the network analysis programs, CytoScape® and Ingenuity Pathway Core Analysis®.

****Results**:**

A microarray study identified eight miRNAs with significantly different expression levels between AAA and controls (adjusted *p* < 0.05). Real-time qRT-PCR assays validated the findings for five of the eight miRNAs. A total of 222 predicted miRNA target genes known to be differentially expressed in AAA based on a prior mRNA microarray study were identified. Bioinformatic analyses revealed that several target genes are involved in apoptosis and activation of T cells.

****Conclusions**:**

Our genome-wide approach revealed several differentially expressed miRNAs in human AAA tissue suggesting that miRNAs play a role in AAA pathogenesis.

## **Background**

Abdominal aortic aneurysm (AAA) is a dilatation of the aorta (>3 cm) that occurs below the renal arteries [1]. In the majority of cases AAA is asymptomatic until it reaches a size that requires surgical intervention due to increased risk of rupture, which is often fatal. The only option for patients diagnosed with AAA ≥55 mm is surgical repair of the aorta, but the risk of surgery must be weighed with the risk of rupture. For patients with smaller AAAs, there is currently no treatment. The most important known risk factors for AAA include smoking, male sex, family history, and advanced age [1,2]. Additionally, biomechanical analyses of AAA demonstrated that there are many factors contributing to aortic wall strength [3]. Previous studies have shown that AAA has a strong genetic component [4], but the biological mechanisms of AAA are not fully understood [2,5]. AAA is characterized by the apoptosis of smooth muscle cells, degradation of the extracellular matrix, a potent inflammatory response, and increased oxidative stress in the abdominal aortic wall [2,5-8]. Infiltration by inflammatory cells may act as mediators that lead to apoptosis of vascular smooth muscle cells [7]. A previous genome-wide mRNA expression study identified a large number of genes with differences in the levels of expression in AAA compared to abdominal aortic tissues from age-and sex-matched controls [9].

MicroRNAs (miRNAs) are a class of small non-coding RNAs, whose primary function is the post-transcriptional regulation of gene expression. miRNAs are incorporated into the RNA induced silencing complex (RISC) and preferentially bind to the 3’ untranslated region (3’UTR) of target mRNA. RISC then inhibits gene expression by either mRNA degradation or by inhibiting translation [10]. miRNAs have been predicted to regulate thousands of target genes [11], which belong to many biological pathways including immune response and apoptosis [12]. Recent studies have demonstrated that miRNAs play roles in several cardiovascular diseases [13].

In the current study, we investigated the expression patterns of microRNAs in AAA as a potential mechanism for the differences in gene expression observed in our prior study [9]. A microarray-based genome-wide screening study was followed by assaying miRNAs individually with real-time quantitative RT-PCR (qRT-PCR). Bioinformatic analyses were carried out to predict gene targets of the miRNAs and analyze their potential roles in AAA.

## **Methods**

### **Human aortic samples**

Full thickness aortic wall tissue specimens were collected from patients undergoing AAA repair operations (n = 41) at the Geisinger Medical Center, Danville, Pennsylvania, USA, or at the Department of Visceral, Thoracic and Vascular Surgery, Technical University of Dresden, Dresden, Germany. Non-aneurysmal aortic samples (n = 12) were collected at autopsies or were obtained from the National Disease Research Interchange (NDRI, Philadelphia, PA; http://www.ndriresource.org). Tissue samples for RNA isolation were stored in RNAlater (Ambion, Austin, TX) or snap-frozen. Table 1 summarizes the demographics of the different groups used in the microarray and qRT-PCR studies. All samples used in the study and information about the donors are listed in Additional file 1: Table S1. The investigation conformed to the principals outlined in the Declaration of Helsinki. AAA patients gave written informed consent for the use of their aortic tissue samples for research. The collection of the human tissues was approved by the Institutional Review Board of Geisinger Clinic, Danville, Pennsylvania, USA, and the Ethics Committee of the Medical Faculty at the Technical University of Dresden, Germany.

**Table 1:** Summary of experimental groups

**Group**	**N**	**Age (Years ± SD)**	**Sex**
**Control – MA**	5	65.4 ± 9.8	3 M, 2 F
**eAAA – MA**	5	64 ± 3.9	3 M, 2 F
**Control - PCR**	7	64.6 ± 4.2	7 M
**eAAA – PCR**	25	70.5 ± 6.1	23 M, 2 F
**rAAA – PCR**	11	71.9 ± 9.1	10 M, 1 F

MA, microarray; PCR, qRT-PCR; M, male; F, female; eAAA, elective repair for AAA; rAAA, ruptured AAA.

For detailed information on donors, see Additional file 1: Table S1.

### **RNA isolation**

RNA was isolated with mirVana™ miRNA Isolation Kit (Ambion Applied Biosystems, Austin, TX). Quality of the RNA samples was assessed by 2100 Bioanalyzer (Agilent Technologies, Inc., Santa Clara, CA).

### **Microarray study**

miRNA expression was compared in AAA (n = 5) and control (n = 5) samples using an Affymetrix GeneChip miRNA 1.0 Array (Santa Clara, CA). The microarray contained 847 miRNAs probes and 922 probes for other small non-coding RNAs. The expression values were computed using the R package Affycoretools version 1.24.0 (available at http://bioconductor.org/) Robust Multivariate Average [14]. miRNAs were identified by calculating the Empirical Bayes Statistics using the R package Limma [15]. Benjamini-Hochberg correction was applied to control the false discovery rate (FDR) [16].

Previously our laboratory generated global mRNA expression profiles for both aneurysmal and non-aneurysmal human infrarenal abdominal aorta [9]. The microarray data can be obtained at the Gene Expression Omnibus (GEO) database (Series# GSE7084; http://www.ncbi.nlm.nih.gov/geo/). We used this data set here for the target gene analysis (see below).

### **Real-time quantitative reverse transcriptase-polymerase chain reaction**

Eight miRNAs (miR-133a, miR-133b, miR-146 a, miR-181a*, miR-204, miR-21, miR-30c-2*, miR-331-3p) which showed significant differences in their levels with an adjusted *p* < 0.05 in the microarray experiment were selected for qRT-PCR validation. TaqMan® MicroRNA Assays for these miRNAs and a small non-coding RNA U6 (Applied Biosystems, Carlsbad, CA) were run according to manufacturer’s recommendation first on RNA from twelve AAA samples from patients undergoing elective repair of an aneurysm and seven control samples that were independent of the microarray study (Table 1 and Additional file 1: Table S1). Next, for the subset of miRNAs with expression medians and variance that warranted further investigation, we expanded the study with an additional thirteen AAA samples from patients undergoing elective repair and eleven AAA samples from patients with aneurysm rupture for a total of seven control, 25 elective repair AAA, and eleven ruptured AAA samples. The relative expression levels of the miRNAs were calculated using the ΔC_T_ method with the expression of the small non-coding RNA U6 as an internal control. The *p* values were calculated using the Wilcoxon rank-sum test using the statistical program R version 2.13.1 (R Foundation for Statistical Computing, Vienna, Austria).

### **Bioinformatic Analyses**

Targets were predicted for qRT-PCR validated miRNAs (miR-133a, miR-133b, miR-331-3p, and miR-204), which were all down regulated in AAA. miR-30c-2* was not included because it is a miRNA* strand; passenger (*) strands of miRNA are usually degraded upon uploading of the miRNA duplex into the RISC complex [17]. The miRNA target prediction databases TargetScan, MirTarget2, and Pictar were queried using the R package RmiR.hsa [18]. The predicted targets were then compared to a list of upregulated genes found in our previous study [9]. In addition, we queried targets of miR-331-3p from TargetScan's non-conserved target prediction dataset (http://www.targetscan.org/) and retrieved gene targets that were conserved across placental mammals.

To evaluate the strength of the binding of miRNAs to their targets, the minimum free energy for miRNA–mRNA hybridization was calculated using program RNAhybrid version 2.1. [19]. The median minimum free energy of hybridization was taken for genes with multiple transcripts. For this analysis the miRNA sequences of qRT-PCR validated miRNAs were retrieved from miRbase version 17 (http://www.mirbase.org). The sequences of the target gene 3’UTR were retrieved from Ensemble Biomart (http://useast.ensembl.org/). CytoScape®, version 2.8.1 software available at http://www.cytoscape.org[20] was used to generate a network showing the miRNA-mRNA connections and indicating the strength of the binding based on the minimum free energy values.

Functional classification of the target genes was carried out with Gene Ontology (GO) analysis using WebGestalt to create a hierarchy of the GO annotations of the predicted targets (http://bioinfo.vanderbilt.edu/webgestalt/). For this procedure, a list of the Entrez IDs for predicted targets that were known to be differentially expressed based on our previous study [9] was uploaded to the web application WebGestalt Gene Set Analysis Toolkit Version 2 [21]. Directed acyclic graphs (DAGs) were generated representing a hierarchical categorization of the significant GO annotations.

Potential target gene interactions were analyzed via networks generated using Ingenuity Pathway Analysis® (IPA) tool version 9.0, (Ingenuity Systems, http://www.ingenuity.com). The four biologically active qRT-PCR-validated miRNAs with their targets were uploaded to IPA. Since IPA combines the targets of mature miRNAs with similar sequences (2–3 nucleotide difference) to miRNA families, experimentally validated targets of miR-133a/miR-133b, miR-211/204, and miR-331-3p were retrieved.

## **Results and Discussions**

**Figure 1:**
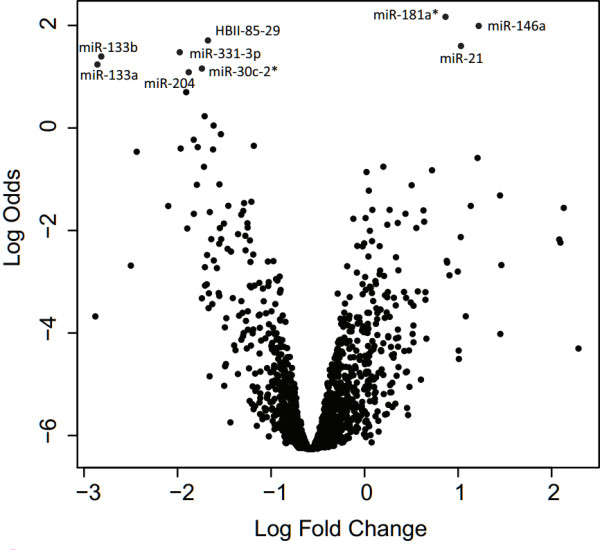
**Volcano plot demonstrating differences in expression levels of miRNAs between AAA and control abdominal aorta based on the microarray study.** The log-fold change is plotted against the log odds of differential expression using the R package limma. The miRNAs with significant differences in expression levels between the AAA (n = 5) and control (n = 5) groups (*p* < 0.05) after Benjamini-Hochberg correction are indicated. Complete lists of nominally significant miRNAs and snoRNAs are shown in Additional file 2: Table S2 and Additional file 3: Table S3, respectively.

A microarray study was performed comparing miRNA expression levels in infrarenal aortic tissue samples between AAA (n = 5) and age- and sex-matched controls (n = 5) (Table 1). The empirical Bayes statistics revealed that out of the 847 miRNAs probes and 922 probes for other small non-coding RNAs, eight miRNAs and one snoRNA had significantly different expression (adjusted *p* < 0.05 after applying Benjamini Hochberg correction; Figure 1) [16]. The three upregulated miRNAs were miR-181a* (MIMAT0000270), miR-146a (MIMAT0000449), and miR-21 (MIMA0000076), while five miRNAs, miR-133b (MIMAT0000770), miR-133a (MIMA000427), miR-331-3p (MIMAT0000760), miR-30c-2* (MIMAT0004550), and miR-204 (MIMA0000265), were significantly down regulated (Figure 1). In addition, HBII-85-29, a small nucleolar RNA, C/D box 116–29, was found to be significantly down regulated (Figure 1). The full lists of the 139 miRNAs and 78 other small non-coding RNAs with expression differences with nominal *p* < 0.05 (no correction for multiple testing) are shown in Additional file 2: Table S2, and Additional file 3: Table S3, respectively.

**Figure 2:**
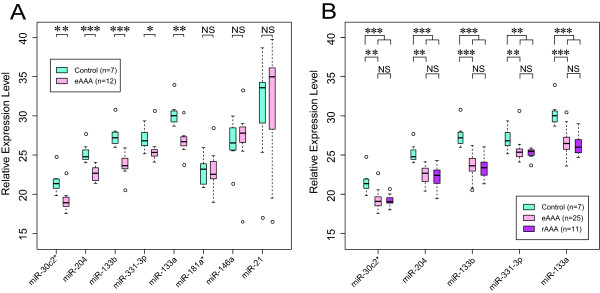
**Validation of microarray results by real-time qRT-PCR in an independent set of samples.A**. Eight miRNAs identified as significantly different between AAA and controls in the microarray study were evaluated by qRT-PCR in an independent set of twelve AAA and seven control samples. **B**. Five down regulated miRNAs validated by qRT-PCR (from panel A), were analyzed using thirteen additional samples from elective AAA repairs (eAAA) and eleven samples from ruptured AAAs (rAAA). The expression levels for each miRNA were adjusted to the expression levels of U6. The *p* values were calculated using the Wilcoxon rank-sum test. Significance between each comparison is shown. NS, not significant; *, *p* < 0.05; **, *p* < 0.001; ***, *p* < 0.0001.

The expression levels of the eight miRNAs identified in the microarray study were initially validated using individual real-time qRT-PCR assays and a new set of twelve AAA and seven control aortic tissue samples (Table 1 and Additional file 1: Table S1). The five down regulated miRNAs showed significantly different expression in AAA tissue compared to the control infrarenal abdominal aorta samples, but the three up regulated miRNAs failed to replicate (Figure 2A). Since it is plausible to hypothesize that AAA initiation, growth and rupture have different molecular mechanisms, we compared the expression levels of the five qRT-PCR-validated miRNAs in ruptured (n = 11) and non-ruptured, electively repaired (n = 25) AAAs (Figure 2B). No significant differences were found between the AAA samples from patients undergoing elective repair operations (eAAA) and those with ruptured AAA (rAAA; Figure 2B). In the combined qRT-PCR analysis including all the 36 AAA tissue samples and seven controls, the differences in expression levels of the five miRNAs, miR-133b, miR-133a, miR-331-3p, miR-30c-2*, and miR-204, between AAA and control groups were highly significant (Figure 2).

We searched the literature for information on miR-133b, miR-133a, miR-204, miR-331-3p, and miR-30c-2*, the five miRNAs with confirmed downregulated expression between AAA and control abdominal aorta. The functions of miR-133b, miR-133a, and miR-204 have been thoroughly examined in a cardiovascular context [22-28], but nothing was known about their role in AAA. A recent study on thoracic aortic dissections [29] found several miRNAs with nominally significant (*p* < 0.05) differences when compared to normal thoracic aorta (summarized in Additional file 2: Table S2). Of the validated miRNAs in the current study only miR-133a and miR-133b differed in expression also in thoracic aortic dissections compared to controls [29]. The differences in the results of these two studies reinforce the distinct nature of these two aortic diseases.

While the current study was under review miR-21 and miR-29b (MIMAT0000100) were identified as potential therapeutic targets in an animal model of aortic aneurysms [30,31]. In addition, miR-21 was shown to be upregulated in human AAA tissue using qRT-PCR [30]. Although miR-21 was upregulated in AAA in our microarray study (Figure 1), it was not validated by qRT-PCR (Figure 2A). The discordant results could be due to differences in ages of the control subjects in the two studies. We did not detect significant differential expression of miR-29b; however, miR-29b-2* was downregulated in our microarray study before FDR correction (Additional file 2: Table S2).

We characterized the putative functions of the miRNAs by identifying genes they are predicted to regulate. Predicted targets for miR-133a, miR-133b, miR-331-3p, and miR-204 were retrieved from the miRNA–mRNA target databases TargetScan, Pictar, and MirTarget2 with the R package RmiR.hsa [18]. There are no predicted target genes for miR-30c-2* in the TargetScan, Pictar, or MirTarget2 data sets [11,32,33]. The list of predicted targets was compared to a list of genes that were previously identified as having altered expression levels in AAA from our microarray-based mRNA expression study [9]. The four downregulated miRNAs miR-133a, miR-133b, miR-331-3p, and miR-204 had 1,836 potential target genes, 222 of which were significantly upregulated in our prior mRNA microarray study (Additional file 4: Table S4) [9], consistent with the proposed regulatory action of the miRNAs.

**Figure 3:**
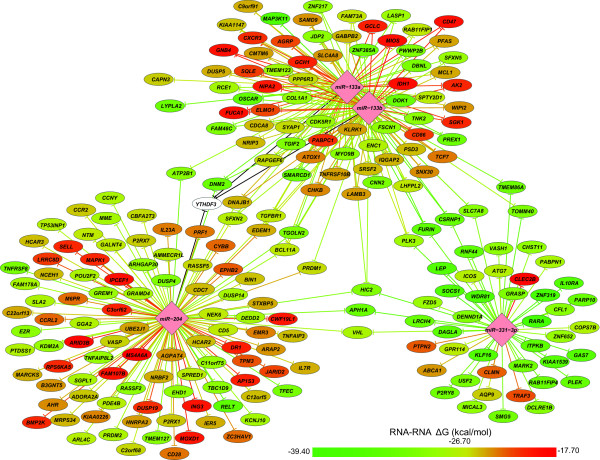
**A network of miRNAs miR-133a, miR-133b, miR-331-3p, miR-204, and their target genes.** miR-30c-2* was not included because it is a miRNA* strand that is usually degraded upon miRNA loading to the RISC complex [17]. Bioinformatic analysis predicted 222 genes (see Additional file 4: Table S4) with upregulated expression in AAA based on a prior microarray study [9] were targets of miR-133a, miR-133b, miR-331, or miR-204. The predicted minimum free energy of the miRNA and target mRNA hybridization from RNAhybrid is shown by the line and node color. Black lines indicate that the 3'UTR sequence was not available from Biomart, and the minimum free energy was not calculated. The figure was generated using CytoScape® [20].

We explored further the miRNA–mRNA interactions with the downloadable version of RNAhybrid program to compute the minimum free energy of the miRNA–mRNA binding [19]. The rationale for this analysis is that interactions with lower predicted minimum free energy are predicted to be more stable and more likely to occur. We ranked the miRNA–target gene interactions and generated a network using CytoScape® to visualize the predicted miRNA–mRNA interactions (Figure 3). There was a redundancy between the predicted targets of miR-133a and miR-133b due to the similarity in their sequences; however, the two base pair difference had an impact on the calculated minimum free energies, suggesting that they may have different affinities for individual target silencing [19]. In general miR-331-3p had the lowest calculated minimum free energy of hybridization to its predicted targets, which suggests it may have stronger binding affinity for its targets.

Several genes were identified as potential targets of two or more of the miRNAs (Figure 3). Four genes (*CSRNP1*, *SLC7AB*, *PLK3*, and *FURIN*) were predicted targets of miR-133a/miR-133b and miR-331-3p. Two genes (*APH1A* and *VHL*) were predicted targets of miR-204 and miR-331-3p. Eight genes (*DNM2, DNAJB1, TGFBR1, TGOLN2, BCL11A, EDEM1, SFXN2, YTHDF3*) were predicted targets of miR-204 and miR-133a/miR-133b. Hypermethylated in cancer 2 (*HIC2)* was the only gene predicted to be targeted by all four miRNAs (Figure 3). Although the function of *HIC2* has not been extensively studied, it is closely related to *HIC1,* which is an important tumor suppressor gene that deactivates repressors of P53 and E2F1 induced senescence [34].

**Figure 4:**
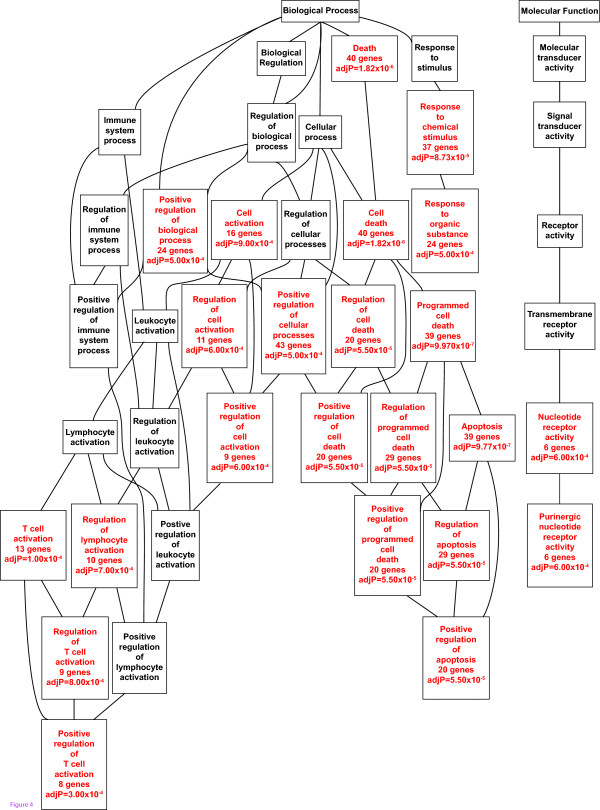
**Biological categories of miRNA target genes.** A DAG of the GO categories of the set of 222 upregulated mRNAs was generated by the web application WebGestalt. Categories shown in red were significant (adjusted *p* < 0.001).

We analyzed the GO terms to assign potential functions to the miRNA targets using the web application, WebGestalt [21]. The results with the most enriched biological processes and molecular functions are shown as a DAG in Figure 4. The most significant biological function was the “*positive regulation of apoptosis*” (Figure 4). It was interesting given the fact that smooth muscle cell apoptosis is a characteristic histological feature of aneurysmal aortic wall in humans. Previous cell culture studies, however, have shown a role for miRNAs in smooth muscle cell proliferation [27,35]. A possible explanation for the contradictory results could be that we are looking at late stages of the human aneurysmal disease requiring surgical intervention, while the previously published cell culture experiments on smooth muscle cells [25] may be more relevant to the initial stages of arterial wall injury. This conclusion is supported by other studies in which mice treated with antagomirs for miR-133a showed cardiomyocyte hypertrophy [25], but knockout mice lacking miR-133a displayed dilated cardiomyopathy with increased apoptosis [22,25]. Another possible explanation is that our results indicate high turnover of vascular smooth muscle cells.

Several target genes with functions in apoptosis were of interest in AAA. Two tumor necrosis factor receptors, *TNFRSF10B* and *TNFRSF8*, were predicted targets of miR-133a/miR-133b and miR-204, respectively. *TNFRSF10B,* also known as death receptor 5, is involved in DR5/FADD/caspase-8 signaling and is an important component of the extrinsic apoptotic pathway [36]. *TNFRSF8*, also known as *CD30,* is involved in NFκB activation and is expressed by activated T and B cells [37]. Tumor protein p53-inducible nuclear protein 1 (*TP53INP1*) is a p53 target gene that responds to multiple types of cellular stress events, including oxidative stress, and promotes cell cycle arrest and apoptosis [38].

Another significant GO term among the target genes included “*T cell activation*” (Figure 4), which is highly relevant finding to AAA, since inflammation is a characteristic of AAA [5], and antigen-independent co-stimulation is a crucial step in T cell activation [39]. *CD28*, *CD86*, and *ICOS*, which are important co-stimulatory molecules, were predicted to be targets of miR-204, miR-133a/miR-133b, and miR-331-3p, respectively [40]. *CD28* and *ICOS* are important receptors of co-stimulatory signals, which are triggered by *ICOSL* in human vascular endothelial cells [39]. *CD86*, which is expressed in antigen presenting cell types including dendritic cells, macrophages, and B cells [41], acts as a ligand to *CD28.* CD86 is not expressed in endothelial cells [40], but its levels are elevated in the plasma [42] of AAA patients. Furthermore, the mRNA levels of *CD86*, *CD80*, *CTLA*, and *ICOS* are elevated in the aortic wall of AAA patients [9].

“*Response to organic substance*” and “*purinergic nucleotide receptor activity*” were additional significant GO terms among the upregulated target genes (Figure 4). Approximately half of the genes annotated in “*Response to organic substance*” were also genes annotated as apoptotic genes. Four of the genes (*DUSP4*, *AQP9*, *SOCS1*, and *PTPN2*) are involved in injury response [43-46]. Genes which were annotated to play roles in responding to organic substance included the niacin receptors, *GPR109A* and *GPR109B*. Niacin has been studied for its potential use based on its anti-inflammatory and anti-atherosclerotic effects of raising HDL [47], although no benefit to patients with clinical disease has been shown to date [48].

**Figure 5:**
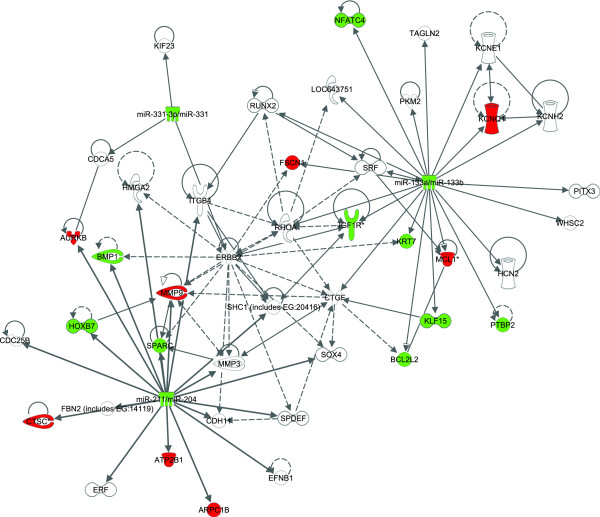
**A network of the interactions of the miRNA target genes.** Ingenuity Pathway Analysis® tool was used to generate the network from experimentally observed miRNA–mRNA interactions of miR-133a/miR-133b, miR-204, and miR-331-3p. Molecules shown in red (increased expression) and green (decreased expression) were identified in our previous microarray study [9]. Solid lines represent direct interactions and dashed lines indirect interactions.

**Figure 6:**
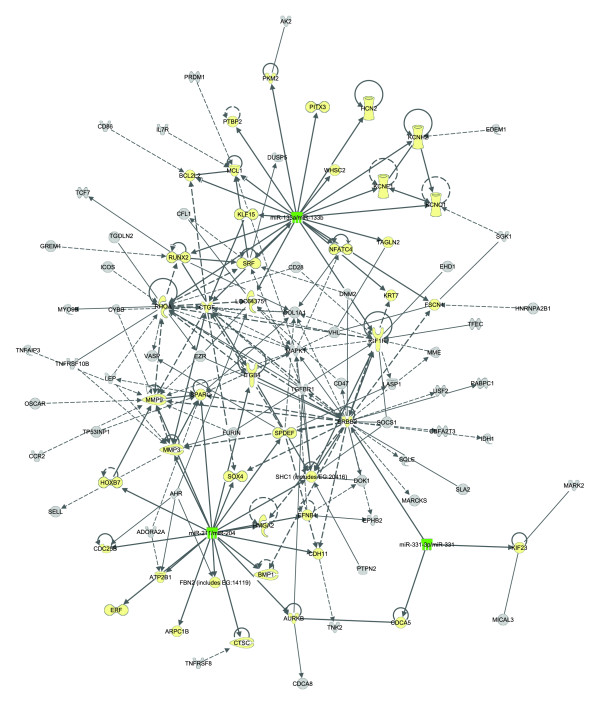
**Expanded network of the validated miRNA–mRNA interactions.** The network was generated using Ingenuity Pathway Analysis® tool. The validated network shown in Figure 5 was expanded to include experimentally validated interactions with our list of the 222 predicted miRNA targets (Additional file 4: Table S4). Green molecules are the four down regulated miRNAs (miR-133a/miR-133b, miR-204, and miR-331-3p), yellow molecules are experimentally verified target genes of the four miRNAs, and grey molecules are predicted targets of the four miRNAs. Solid lines represent direct connections and dashed lines represent indirect connections

Ingenuity Systems® Pathway Analysis tool was used to generate a network from 45 experimentally verified interactions of the four biologically active, validated, down regulated miRNAs (miR-133a, miR-133b, miR-331-3p, and miR-204) (Figure 5). We further explored the regulation of the targets by examining the interactions of the validated targets with the predicted miRNA targets (Figure 6), and found that 54 of the predicted miRNA targets interacted with the experimentally validated miRNA targets. The interaction networks (Figures 3, 5, and 6) demonstrate a complex role for miRNAs in AAA. Since miRNAs usually inhibit mRNA expression of their target genes [10], we expected that target mRNAs of the miRNAs down regulated in AAA would be upregulated; several gene targets exhibited expression levels consistent with this expectation (shown in red in Figure 5). For example, *MMP9* was identified as a target of miR-204 [49]. This finding is highly relevant to AAA pathogenesis, since the decreased level of miR-204 could contribute to the increased mRNA and protein expression level of MMP9 seen in human AAA tissue (Figures 5 and 6), and thereby increase the degradation of the extracellular matrix in AAA [50].

There were also several genes known to be regulated by these miRNAs whose expression was decreased in AAA (shown in green in Figure 5) [9]. Most of these genes have proliferative and anti-apoptotic functions based on our literature search. For example, in cell culture experiments, miR-133a/miR-133b down regulation is associated with a switch in vascular smooth muscle cells to a proliferative phenotype [27]. miR-133a regulates the expression of a gene called nuclear factor of activated T cells, calcineurin-dependent-4 (*NFATc4*/NFAT3), which is a ubiquitously expressed member of the NFAT transcription factor family and is involved in cell proliferation. *NFATC4* mediates the effect of miR-133a in increasing cell proliferation in cardiomyocyte hypertrophy *in vivo*[24]*,* but its expression is decreased in AAA [9]. One potential explanation for the down regulation of *NFATC4* is competition with other biological pathways. *HOXA9* indirectly promotes *NFATC4* expression [51,52]. The expression of the members of the *HOXA* family is decreased in AAA [53]. Also, in hypertrophy *NFATC4* is regulated by calcium mediated response of angiotensin II, endothelin, and norepinephrine binding to their receptors [54], but in AAA the expression of these receptors is down regulated [9]. *KLF15* is also a target of miR-133a/miR-133b [23], and its expression is reduced in both mouse aneurysm models and human AAA [9,55]. These findings suggest that multiple competing regulators are important in AAA, and the phenotype is a result of complex interactions between regulatory molecules with different functions.

Our study has several limitations. One limitation is the use of end-stage disease human tissue, since it is plausible to collect human aortic aneurysmal samples only from AAAs large enough to require surgical intervention or from ruptured aortas. Additionally, since the study is observational, it is not possible to differentiate between cause and consequence, which would require intervention in a model system. It is also possible that the differences in mRNA and miRNA expression are merely reflective of the changes in the aortic wall architecture in AAA. The histological characterization of the aortic wall in AAA to “inflammatory”, “active” and “amorphous regions” has been proposed [56,57]; the regions may, however, overlap and do not necessarily show a clear progression of the disease. Based on histological and immunohistochemical analyses in our previous studies [58,59], the samples were from the so called “active region” of the AAAs.

## **Conclusions**

Our genome-wide study followed by qRT-PCR validation identified five miRNAs with significantly downregulated expression in AAA aortic tissue from a control group of human infrarenal aortic tissues. Bioinformatic analysis indicated that miR-133a, miR-133b, miR-331-3p, and miR-204 target apoptotic genes, which may play a role in the loss of vascular smooth muscle cells in AAA. The miRNAs are also involved in the activation of the immune cells and the alteration of their response to chemical signaling. Taken together, the results provide strong evidence for an important regulatory function of miRNAs in vascular remodeling of the aorta.

## **Abbreviations**

AAA: Abdominal Aortic Aneurysm; DAG: Directed Acyclic Graph; FDR: False Discovery Rate; GEO: Gene Expression Omnibus; IPA®: Ingenuity Pathway Analysis®; miRNA: microRNA; NDRI: National Disease Research Interchange; RT-PCR: Reverse Transcriptase-PCR; RISC: RNA induced silencing complex; qRT-PCR: quantitative Reverse Transcriptase-PCR; snoRNA: small non-coding RNA; 3’UTR: 3’ untranslated region.

## **Competing interests**

The authors declare that they have no competing interests.

## **Authors' contributions**

MCP designed experiments, analyzed data, carried out bioinformatic analyses, and drafted the manuscript. KD prepared RNA samples for microarray and qRT-PCR, and ran the qRT-PCR assays. GG, IH, JRE and DPF recruited patients, obtained tissue samples from cases and controls, verified clinical information and critically reviewed the manuscript. CMS carried out the microarray analyses. TCP and DJC contributed to the experimental design and data analysis, and critically reviewed the manuscript. JLG recruited patients, and obtained tissue samples. GT contributed to the experimental design, statistical analysis, computational aspects, as well as drafting and editing of the manuscript. HK contributed to the experimental design, data analysis, drafting and editing of the manuscript, and obtained funding for the study. All authors read and approved the final manuscript.

## Pre-publication history

The pre-publication history for this paper can be accessed here:

http://www.biomedcentral.com/1755-8794/5/25/prepub

## Supplementary Material

Additional file 1 **Table S1.** Samples used in microarray and real time qRT-PCR experiments. Click here for file

Additional file 2 **Table S2.** List of the miRNAs which were found to have significantly different (nominal p < 0.05) expression in AAA (n = 5) compared to control tissue (n = 5). Click here for file

Additional file 3 **Table S3.** List of other small RNAs with significantly different (nominal p < 0.05) expression in AAA (n = 5) compared to controls (n = 5). Click here for file

Additional file 4 **Table S4.** A list of predicted target genes for miR-133a/miR-133b, miR-204, and miR-331-3p that were also upregulated in our prior microarray study. Click here for file
