# Supplementary material for: MicroRNA expression signature in human abdominal aortic aneurysms
Source: BMC Med Genomics. 2012 Jun 15;5:25. doi: 10.1186/1755-8794-5-25 (PMC3507654; doi:10.1186/1755-8794-5-25)
Supplement: Additional file 1 — Table S1. Samples used in microarray and real time qRT-PCR experiments. [file 1755-8794-5-25-S1.pdf]

Additional File 1

Table S1. Samples used in microarray and real time qRT-PCR experiments

Case ID	Classification	Sex	Age (Years)	Microarray Study	Real Time RT-PCR	Cause of Death
NDRI57179	Control	F	57	X		Respiratory failure
ME0503	Control	M	54	X		Cardiac arrest
ME0501	Control	F	69	X		Head trauma
ME0205	Control	M	78	X		Cardiac arrest
NDRI57054	Control	M	69	X		Cardiac arrest
AAA0007	Elective Repair	F	65	X		
AAA0012	Elective Repair	M	70	X		
AAA200210	Elective Repair	M	60	X		
AAA200305	Elective Repair	M	64	X		
AAA0011	Elective Repair	F	61	X		
NDRI66274	Control	M	61		X	Cardiac arrest
NDRI66279	Control	M	64		X	Pancreatic cancer
NDRI66324	Control	M	67		X	Metastatic lung cancer
NDRI57110	Control	M	69		X	Cardiac arrest
NDRI66747	Control	M	57		X	Cardiac arrest
NDRI66799	Control	M	67		X	Respiratory
NDRI66862	Control	M	67		X	Respiratory
AAA200219	Elective Repair	M	67		X	
AAA0005	Elective Repair	M	64		X	
AAA0006	Elective Repair	F	81		X	
AAA200212	Elective Repair	M	73		X	
AAA200214	Elective Repair	M	60		X	
AAA0001	Elective Repair	M	67		X	
AAA200305	Elective Repair	M	64		X	
AAA0015	Elective Repair	M	66		X	
AAA0014	Elective Repair	M	75		X	
AAA0019	Elective Repair	M	66		X	
AAA0017	Elective Repair	M	78		X	
AAA0026	Elective Repair	M	67		X	
GV333	Elective Repair	M	75		X	
GV214	Elective Repair	M	72		X	
GV256	Elective Repair	M	81		X	
GV290	Elective Repair	M	67		X	
GV172	Elective Repair	M	66		X	
GV242	Elective Repair	M	68		X	
GV266	Elective Repair	M	77		X	
GV231	Elective Repair	M	78		X	
GV257	Elective Repair	M	70		X	
GV261	Elective Repair	M	72		X	
GV293	Elective Repair	F	76		X	
GV305	Elective Repair	M	72		X	
GV289	Elective Repair	M	60		X	
752189	Ruptured Aneurysm	M	51		X	
823701	Ruptured Aneurysm	M	75		X	
843624	Ruptured Aneurysm	M	78		X	
GV286	Ruptured Aneurysm	M	74		X	
688589	Ruptured Aneurysm	M	78		X	
779967	Ruptured Aneurysm	M	71		X	
806903	Ruptured Aneurysm	M	64		X	
GV236	Ruptured Aneurysm	M	75		X	
GV283	Ruptured Aneurysm	F	86		X	
GV292	Ruptured Aneurysm	M	67		X	
GV251	Ruptured Aneurysm	M	72		X	

All individuals were white.

All samples were taken from the infrarenal region of the abdominal aorta. Control samples were obtained at autopsies and causes of death are given. Elective repair and ruptured aneurysm samples were taken during AAA repair operations.

The summary statistics for the microarray experiment are:

- AAA group (n = 5): Mean age = 64 +/- 3.9; Median = 64; Male:Female = 3:2

- Control group (n = 5): Mean age = 65.4 +/- 9.8; Median = 69; Male:Female = 3:2
- Comparison of ages in the study groups: *P* = 1.00 (Wilcoxon rank-sum test)

The summary statistics for the real time qRT-PCR:

- Elective repair group (n = 25): Mean age = 70.49 +/- 6.1; Median = 70; Male:Female = 23:2
- Ruptured aneurysm group (n = 11): Mean age = 71.9 +/- 9.1; Median = 74; Male:Female = 10:1
- Control group (n = 7): Mean age = 64.6 +/- 4.2; Median = 67; Male:Female = 7:0
- First qRT-PCR validation experiment: Comparison of ages for control group (n = 7) and aneurysm group (n = 12): *P* = 0.34 (Wilcoxon rank-sum test)
- Comparison of ages for control group (n = 7) and elective repair group (n = 25): *P* = 0.049 (Wilcoxon rank-sum test)
- Comparison of ages for elective repair group (n = 25) and ruptured aneurysm group (n = 11): *P* = 0.42 (Wilcoxon rank-sum test)
