# Supplementary material for: MicroRNA expression signature in human abdominal aortic aneurysms
Source: BMC Med Genomics. 2012 Jun 15;5:25. doi: 10.1186/1755-8794-5-25 (PMC3507654; doi:10.1186/1755-8794-5-25)
Supplement: Additional file 2 — Table S2. List of the miRNAs which were found to have significantly different (nominal p < 0.05) expression in AAA (n = 5) compared to control tissue (n = 5). [file 1755-8794-5-25-S2.pdf]

Additional File 2
Table S2. List of the miRNAs which were found to have significantly different (nominal P < 0.05) expression in AAA (n = 5) compared to control tissue (n = 5)

miRNA	Fold Change (log_2)	*P* Value	Adjusted *P* Value	Expression in TAD*
hsa-miR-181a*	1.40	6.72E-05	0.04	
hsa-miR-146a	2.24	8.20E-05	0.04	D
hsa-miR-21	2.28	1.27E-04	0.04	
hsa-miR-331-3p	1.16	1.45E-04	0.04	
hsa-miR-133b	1.30	1.59E-04	0.04	C
hsa-miR-133a	1.33	1.89E-04	0.04	C
hsa-miR-30c-2*	1.13	2.07E-04	0.04	
hsa-miR-204	1.04	2.23E-04	0.04	
hsa-miR-193a-5p	0.61	3.39E-04	0.06	C
hsa-miR-99b	1.21	5.60E-04	0.09	
hsa-miR-29b-2*	1.39	6.78E-04	0.10	
hsa-miR-1287	1.86	1.03E-03	0.11	
hsa-miR-1296	1.22	1.05E-03	0.11	
hsa-miR-504	0.71	1.09E-03	0.11	
hsa-miR-149	1.52	1.16E-03	0.11	
hsa-miR-650	0.72	1.31E-03	0.12	
hsa-miR-21*	1.25	1.69E-03	0.13	
hsa-miR-887	0.93	2.26E-03	0.15	
hsa-miR-608	0.67	2.28E-03	0.15	
hsa-miR-1275	0.97	2.81E-03	0.17	
hsa-miR-10a	1.32	3.28E-03	0.17	
hsa-miR-146b-5p	0.78	3.47E-03	0.17	
hsa-miR-193b	0.70	3.48E-03	0.17	
hsa-miR-297	1.06	3.61E-03	0.17	
hsa-miR-503	0.96	3.80E-03	0.17	
hsa-miR-30c	0.89	3.84E-03	0.17	D
hsa-miR-342-5p	0.70	4.06E-03	0.17	
hsa-miR-328	0.85	4.07E-03	0.17	
hsa-miR-346	0.61	4.77E-03	0.18	
hsa-miR-423-3p	1.11	4.95E-03	0.18	
hsa-miR-618	1.04	5.36E-03	0.18	
hsa-miR-424*	0.40	5.40E-03	0.18	
hsa-miR-339-5p	1.92	5.42E-03	0.18	
hsa-miR-143*	1.13	5.45E-03	0.18	
hsa-miR-623	1.00	5.71E-03	0.18	
hsa-miR-361-5p	0.45	6.11E-03	0.19	
hsa-miR-339-3p	0.55	6.32E-03	0.20	
hsa-miR-647	1.11	6.49E-03	0.20	
hsa-let-7e*	1.13	6.76E-03	0.20	C
hsa-miR-30d	0.60	6.77E-03	0.20	
hsa-miR-27a*	0.45	6.80E-03	0.20	
hsa-miR-130b	0.64	7.03E-03	0.20	
hsa-miR-23a*	0.51	7.25E-03	0.20	
hsa-miR-137	0.32	7.78E-03	0.20	
hsa-miR-1291	0.33	8.23E-03	0.21	
hsa-miR-30a*	1.09	8.47E-03	0.21	C
hsa-miR-28-5p	0.98	8.68E-03	0.21	
hsa-miR-196a	0.83	9.21E-03	0.22	
hsa-miR-24-1*	1.16	9.30E-03	0.22	
hsa-miR-1280	0.74	1.04E-02	0.23	
hsa-miR-146b-3p	0.50	1.05E-02	0.23	C
hsa-miR-614	1.05	1.05E-02	0.23	
hsa-miR-150	0.38	1.14E-02	0.24	
hsa-miR-206	1.09	1.15E-02	0.24	
hsa-miR-28-3p	0.67	1.19E-02	0.24	
hsa-miR-125a-5p	0.42	1.21E-02	0.25	
hsa-miR-660	0.73	1.26E-02	0.25	
hsa-miR-32	0.48	1.27E-02	0.25	
hsa-miR-595	0.54	1.30E-02	0.25	
hsa-miR-31	0.55	1.40E-02	0.27	
hsa-miR-199a-3p	0.77	1.45E-02	0.27	
hsa-miR-499-3p	2.30	1.61E-02	0.28	
hsa-miR-10b	0.90	1.63E-02	0.28	
hsa-miR-181a	0.33	1.67E-02	0.28	
hsa-miR-1279	0.69	1.68E-02	0.28	
hsa-miR-125b-1*	0.27	1.68E-02	0.28	
hsa-miR-197	0.45	1.72E-02	0.28	
hsa-miR-100	0.54	1.72E-02	0.28	C
hsa-miR-140-3p	0.40	1.74E-02	0.28	C
hsa-miR-301b	0.61	1.79E-02	0.29	
hsa-miR-324-5p	0.67	1.83E-02	0.29	

miRNA	Fold Change (log_2)	*P* Value	Adjusted *P* Value	Expression in TAD*
hsa-miR-199b-3p	0.39	1.88E-02	0.29	
hsa-miR-182*	0.62	1.88E-02	0.29	
hsa-miR-148a	0.45	1.94E-02	0.29	
hsa-miR-610	0.39	1.96E-02	0.29	
hsa-miR-138-1*	0.72	1.97E-02	0.29	C
hsa-miR-1305	-1.44	1.99E-02	0.29	
hsa-miR-1247	-1.80	2.02E-02	0.29	D
hsa-miR-629*	-1.61	2.05E-02	0.29	
hsa-miR-612	-1.79	2.10E-02	0.30	
hsa-miR-184	-1.30	2.12E-02	0.30	
hsa-miR-188-5p	-1.08	2.21E-02	0.30	
hsa-miR-593*	-2.03	2.21E-02	0.30	
hsa-miR-744	-1.71	2.22E-02	0.30	C
hsa-miR-933	-2.71	2.24E-02	0.30	
hsa-miR-29c*	-1.21	2.36E-02	0.31	
hsa-miR-519b-3p	-1.01	2.37E-02	0.31	
hsa-miR-1183	-1.21	2.43E-02	0.31	
hsa-miR-519b-5p	-1.13	2.48E-02	0.32	
hsa-miR-572	-0.63	2.51E-02	0.32	
hsa-miR-545	-1.60	2.51E-02	0.32	
hsa-miR-1322	-2.66	2.57E-02	0.32	
hsa-miR-548c-5p	-0.66	2.60E-02	0.32	
hsa-miR-374b	-2.67	2.73E-02	0.34	
hsa-miR-200c*	-0.74	2.80E-02	0.34	
hsa-miR-330-3p	-1.46	2.90E-02	0.35	
hsa-miR-141*	-2.04	2.90E-02	0.35	
hsa-miR-1182	-0.94	2.98E-02	0.35	
hsa-miR-574-3p	-0.74	3.00E-02	0.35	
hsa-miR-335*	-1.57	3.00E-02	0.35	
hsa-miR-126	-1.48	3.01E-02	0.35	
hsa-miR-744*	-0.58	3.03E-02	0.35	
hsa-miR-30a	-0.70	3.11E-02	0.35	C
hsa-miR-425*	-0.56	3.16E-02	0.35	
hsa-miR-99b*	-0.59	3.18E-02	0.35	
hsa-miR-1261	-1.15	3.18E-02	0.35	
hsa-miR-122	-0.83	3.23E-02	0.35	
hsa-miR-187	-1.23	3.23E-02	0.35	
hsa-miR-629	-0.56	3.26E-02	0.35	
hsa-miR-92a-1*	-0.80	3.33E-02	0.35	
hsa-miR-484	-0.89	3.35E-02	0.35	
hsa-miR-223*	-1.02	3.38E-02	0.35	
hsa-miR-581	-1.07	3.44E-02	0.35	
hsa-miR-532-3p	-0.37	3.54E-02	0.36	
hsa-miR-379*	-0.91	3.61E-02	0.36	
hsa-let-7i	-1.10	3.61E-02	0.36	
hsa-miR-625*	-0.75	3.75E-02	0.37	
hsa-miR-1284	-0.81	3.76E-02	0.37	C
hsa-miR-384	-0.62	3.77E-02	0.37	
hsa-miR-621	-0.71	3.79E-02	0.37	
hsa-miR-148b	-0.42	3.89E-02	0.37	
hsa-miR-877*	-1.66	4.05E-02	0.38	
hsa-miR-874	-0.85	4.08E-02	0.38	
hsa-miR-9	-0.79	4.09E-02	0.38	
hsa-miR-145*	-0.32	4.16E-02	0.38	
hsa-miR-518c	-0.52	4.22E-02	0.38	
hsa-miR-181a-2*	-0.50	4.24E-02	0.38	
hsa-miR-593	-0.70	4.39E-02	0.39	
hsa-miR-337-3p	-0.34	4.46E-02	0.39	
hsa-miR-574-5p	-1.10	4.49E-02	0.39	
hsa-miR-566	-0.61	4.52E-02	0.39	
hsa-miR-126*	-0.62	4.57E-02	0.39	
hsa-miR-151-3p	-0.44	4.59E-02	0.39	C
hsa-miR-548a-3p	-0.57	4.64E-02	0.39	
hsa-miR-138	-0.60	4.64E-02	0.39	
hsa-miR-514	-1.09	4.64E-02	0.39	
hsa-miR-768-5p	-2.03	4.85E-02	0.40	D
hsa-miR-1231	-0.83	4.86E-02	0.40	

Includes the log_2 fold change, as well as raw and Benjamini-Hochberg adjusted *P* values. We have also indicated if the miRNA was differentially expressed in another microarray-based miRNA study on patients with thoracic aortic dissection (TAD)* and controls. C, concordant result, differentially expressed in TAD and in the same direction (up/downregulated) as in our study; D, disconcordant, differentially expressed in TAD but was in the opposite direction from our study.

***Liao M, Zou S, Weng J, Hou L, Yang L, Zhao Z, Bao J, and Jing Z.** A microRNA profile comparison between thoracic aortic dissection and normal thoracic aorta indicates the potential role of microRNAs in contributing to thoracic aortic dissection pathogenesis. *J Vasc Surg* 53: 1341-1349 e1343, 2011.
