# Supplementary material for: MicroRNA expression signature in human abdominal aortic aneurysms
Source: BMC Med Genomics. 2012 Jun 15;5:25. doi: 10.1186/1755-8794-5-25 (PMC3507654; doi:10.1186/1755-8794-5-25)
Supplement: Additional file 3 — Table S3. List of other small RNAs with significantly different (nominal p < 0.05) expression in AAA (n = 5) compared to controls (n = 5). [file 1755-8794-5-25-S3.pdf]

Additional File 3

Table S3. List of other small RNAs with significantly different (nominal P < 0.05) expression in AAA (n = 5) compared to controls (n = 5)

Small RNA	Fold Change (log_2)	*P* Value	Adjusted *P* Value
HBII-85-29	-1.10	1.13E-04	0.04
HBII-52-44	-0.96	8.11E-04	0.11
HBII-85-3	-1.25	9.07E-04	0.11
HBII-85-29	-1.04	1.11E-03	0.11
U101	0.78	1.57E-03	0.13
HBII-85-15	-1.14	1.58E-03	0.13
U24	0.60	1.75E-03	0.13
HBII-85-21	-0.97	2.25E-03	0.15
U71c	0.62	2.55E-03	0.16
HBII-85-25_s	-0.63	3.19E-03	0.17
HBII-52-32	-0.88	3.46E-03	0.17
HBI-61	0.85	3.75E-03	0.17
U96b	0.66	3.76E-03	0.17
HBII-85-17	-1.08	3.93E-03	0.17
HBII-85-14	-0.74	4.13E-03	0.17
U71c	0.59	4.43E-03	0.18
ENSG00000199452	0.46	4.49E-03	0.18
U49A	0.93	4.88E-03	0.18
HBII-85-22	-0.68	4.92E-03	0.18
U18C	0.82	5.07E-03	0.18
HBII-85-2	-0.65	6.93E-03	0.2
ACA51	0.57	7.35E-03	0.2
HBII-85-11	-0.98	7.42E-03	0.2
U37	0.50	7.76E-03	0.2
ENSG00000212175	0.56	7.81E-03	0.2
U15A	0.62	9.58E-03	0.23
U49A	0.91	9.73E-03	0.23
HBII-52-9	-0.46	1.06E-02	0.23
HBII-85-8	-0.64	1.07E-02	0.23
U50	1.46	1.08E-02	0.23
ENSG00000212182	0.39	1.16E-02	0.24
HBII-296B	0.49	1.33E-02	0.26
U70	0.74	1.37E-02	0.26
ACA24	0.79	1.42E-02	0.27
HBII-85-27	-0.33	1.44E-02	0.27
ENSG00000207171	-0.37	1.51E-02	0.28
ENSG00000212132	-0.36	1.56E-02	0.28
U46	0.69	1.57E-02	0.28
HBII-85-5	-0.65	1.73E-02	0.28
ENSG00000207002	0.63	1.76E-02	0.29
HBII-180B	0.64	1.81E-02	0.29
U27	1.00	1.96E-02	0.29
U79	0.66	2.00E-02	0.29
U107	-0.98	2.01E-02	0.29
14qII-11	0.29	2.03E-02	0.29
U28	0.42	2.17E-02	0.3
ACA24_s	0.82	2.19E-02	0.3
U25	1.23	2.30E-02	0.31
snR38C	0.67	2.35E-02	0.31
U17b	0.70	2.73E-02	0.34
U51	0.75	2.81E-02	0.34
ENSG00000212565	0.36	2.99E-02	0.35
U59B	0.61	3.00E-02	0.35
ENSG00000201853	-0.30	3.16E-02	0.35
U67	0.50	3.29E-02	0.35
ACA22_s	0.52	3.30E-02	0.35
14qII-30	0.36	3.39E-02	0.35
U19	0.41	3.39E-02	0.35
U57	0.71	3.42E-02	0.35
HBII-52-10	-0.32	3.51E-02	0.36
ENSG00000201619	0.61	3.76E-02	0.37
ENSG00000212139	0.33	3.77E-02	0.37
14qII-11	0.37	3.78E-02	0.37
ENSG00000207274	0.40	3.88E-02	0.37
HBII-336	0.56	3.95E-02	0.38
ENSG00000212581	0.35	4.00E-02	0.38

Small RNA	Fold Change (log_2)	*P* Value	Adjusted *P* Value
ENSG00000207177	0.44	4.02E-02	0.38
HBII-85-23	-0.92	4.04E-02	0.38
U21	0.56	4.12E-02	0.38
U34	0.93	4.23E-02	0.38
snR38B	0.55	4.32E-02	0.39
ACA67	0.42	4.39E-02	0.39
HBII-85-24	-0.72	4.54E-02	0.39
U71d	0.61	4.54E-02	0.39
HBII-85-13	-0.37	4.57E-02	0.39
U104	0.71	4.74E-02	0.4
mgh28S-2411	0.83	4.97E-02	0.41

Includes the log_2 fold change, as well as raw and Benjamini-Hochberg adjusted *P* values.
