# Supplementary material for: MicroRNA expression signature in human abdominal aortic aneurysms
Source: BMC Med Genomics. 2012 Jun 15;5:25. doi: 10.1186/1755-8794-5-25 (PMC3507654; doi:10.1186/1755-8794-5-25)
Supplement: Additional file 4 — Table S4. A list of predicted target genes for miR-133a/miR-133b, miR-204, and miR-331-3p that were also upregulated in our prior microarray study. [file 1755-8794-5-25-S4.pdf]

Additional File 4

Table S4. A list of predicted target genes for miR-133a/miR-133b, miR-204, and miR-331-3p that were also upregulated in our prior microarray study*

Gene Symbol	GeneID	Description
AADACL1	57552	arylacetamide deacetylase-like 1
ABCA1	19	ATP-binding cassette, sub-family A (ABC1), member 1
ADORA2A	135	adenosine A2a receptor
AGPAT4	56895	1-acylglycerol-3-phosphate O-acyltransferase 4 (lysophosphatidic acid acyltransferase, delta)
AGRP	181	agouti related protein homolog (mouse)
AHR	196	aryl hydrocarbon receptor
AK2	204	adenylate kinase 2
AP1S3	130340	adaptor-related protein complex 1, sigma 3 subunit
APH1A	51107	anterior pharynx defective 1 homolog A (C. elegans)
AQP9	366	aquaporin 9
ARHGAP30	257106	Rho GTPase activating protein 30
ARID3B	10620	AT rich interactive domain 3B (BRIGHT- like)
ARL4C	10123	ADP-ribosylation factor-like 4C
ATG7	10533	Autophagy-related 7 homolog (S. cerevisiae)
ATOX1	475	ATX1 antioxidant protein 1 homolog (yeast)
ATP2B1	490	ATPase, Ca++ transporting, plasma membrane 1
AXUD1	64651	AXIN1 up-regulated 1
B3GNT5	84002	UDP-GlcNAc:betaGal beta-1,3-N-acetylglucosaminyltransferase 5
BCL11A	53335	B-cell CLL/lymphoma 11A (zinc finger protein)
BIN1	274	bridging integrator 1
BMP2K	55589	BMP2 inducible kinase
C10orf6	55719	chromosome 10 open reading frame 6
C10orf9	219771	chromosome 10 open reading frame 9
C11orf11	747	chromosome 11 open reading frame 11
C12orf5	57103	chromosome 12 open reading frame 5
C22orf13	83606	chromosome 22 open reading frame 13
C3orf62	375341	chromosome 3 open reading frame 62
C9orf91	203197	chromosome 9 open reading frame 91
CAPN3	825	calpain 3, (p94)
CBFA2T3	863	core-binding factor, runt domain, alpha subunit 2; translocated to, 3
CCR2	1231	chemokine (C-C motif) receptor 2
CCRL2	9034	chemokine (C-C motif) receptor-like 2
CD28	940	CD28 antigen (Tp44)
CD47	961	CD47 antigen (Rh-related antigen, integrin-associated signal transducer)
CD5	921	CD5 antigen (p56-62)
CD86	942	CD86 antigen (CD28 antigen ligand 2, B7-2 antigen)
CDC7	8317	CDC7 cell division cycle 7 (S. cerevisiae)
CDCA8	55143	cell division cycle associated 8
CDK5R1	8851	cyclin-dependent kinase 5, regulatory subunit 1 (p35)
CENTD1	116984	centaurin, delta 1
CFL1	1072	cofilin 1 (non-muscle)
CHST11	50515	Carbohydrate (chondroitin 4) sulfotransferase 11
CLEC2B	9976	C-type lectin domain family 2, member B
CLMN	79789	calmin (calponin-like, transmembrane)
CMTM6	54918	CKLF-like MARVEL transmembrane domain containing 6
CNN2	1265	calponin 2
COL1A1	1277	collagen, type I, alpha 1
COPS7B	64708	COP9 constitutive photomorphogenic homolog subunit 7B (Arabidopsis)
CWF19L1	55280	CWF19-like 1, cell cycle control (S. pombe)
CXCR3	2833	chemokine (C-X-C motif) receptor 3
CYBB	1536	cytochrome b-245, beta polypeptide (chronic granulomatous disease)
DAGLA	747	Diacyglycerol lipase, alpha
DBNL	28988	drebrin-like
DCLRE1B	64858	DNA cross-link repair 1B (PSO2 homolog, S. cerevisiae)
DEDD2	162989	death effector domain containing 2
DENND1A	57706	DENN/MADD domain containing 1A
DIP	23151	death-inducing-protein

Gene Symbol	GeneID	Description
DNAJB1	3337	DnaJ (Hsp40) homolog, subfamily B, member 1
DNM2	1785	dynamin 2
DOK1	1796	docking protein 1, 62kDa (downstream of tyrosine kinase 1)
DR1	1810	down-regulator of transcription 1, TBP-binding (negative cofactor 2)
DUSP14	11072	dual specificity phosphatase 14
DUSP19	142679	dual specificity phosphatase 19
DUSP4	1846	dual specificity phosphatase 4
DUSP5	1847	dual specificity phosphatase 5
EDEM1	9695	ER degradation enhancer, mannosidase alpha-like 1
EHD1	10938	EH-domain containing 1
ELMO1	9844	engulfment and cell motility 1
EMR3	84658	egf-like module containing, mucin-like, hormone receptor-like 3
ENC1	8507	ectodermal-neural cortex (with BTB-like domain)
EPHB2	2048	EPH receptor B2
FAM107B	83641	family with sequence similarity 107, member B
FAM46C	54855	family with sequence similarity 46, member C
FAM73A	374986	family with sequence similarity 73, member A
FBXL11	22992	F-box and leucine-rich repeat protein 11
FLJ20323	54468	hypothetical protein FLJ20323
FN5	56935	FN5 protein
FSCN1	6624	fascin homolog 1, actin-bundling protein (Strongylocentrotus purpuratus)
FUCA1	2517	fucosidase, alpha-L- 1, tissue
FURIN	5045	furin (paired basic amino acid cleaving enzyme)
FZD5	7855	frizzled family receptor 5
GALNT4	8693	UDP-N-acetyl-alpha-D-galactosamine:polypeptide N-acetylgalactosaminyltransferase 4 (GalNAc-T4)
GCH1	2643	GTP cyclohydrolase 1 (dopa-responsive dystonia)
GCLC	2729	glutamate-cysteine ligase, catalytic subunit
GGA2	23062	golgi associated, gamma adaptin ear containing, ARF binding protein 2
GNB4	59345	guanine nucleotide binding protein (G protein), beta polypeptide 4
GPR109A	338442	G protein-coupled receptor 109A
GPR109B	8843	G protein-coupled receptor 109B
GPR114	221188	G protein-coupled receptor 114
GRASP	160622	GRP1 (general receptor for phosphoinositides 1)-associated scaffold protein
GREM1	26585	gremlin 1, cysteine knot superfamily, homolog (Xenopus laevis)
HIC2	23119	hypermethylated in cancer 2
HNRPA2B1	3181	heterogeneous nuclear ribonucleoprotein A2/B1
HNT	50863	Neurotrimin
ICOS	29851	inducible T-cell co-stimulator
IDH1	3417	isocitrate dehydrogenase 1 (NADP+), soluble
IER5	51278	immediate early response 5
IL10RA	3587	Interleukin 10 Receptor, alpha
IL23A	51561	interleukin 23, alpha subunit p19
IL7R	3575	interleukin 7 receptor
ING3	54556	inhibitor of growth family, member 3
IQGAP2	10788	IQ motif containing GTPase activating protein 2
ITPKB	3707	inositol trisphospphate 3-kinase B
JARID2	3720	Jumonji, AT rich interactive domain 2
JDP2	122953	jun dimerization protein 2
KCNJ10	3766	potassium inwardly-rectifying channel, subfamily J, member 10
KIAA0226	9711	KIAA0226
KIAA1539	80256	KIAA1539
KLF16	83855	Kruppel-like factor 16
KLRK1	22914	killer cell lectin-like receptor subfamily K, member 1
LAMB3	3914	laminin, beta 3
LASP1	3927	LIM and SH3 protein 1
LCHN	57189	LCHN protein
LEP	3952	leptin (obesity homolog, mouse)
LHFPL2	10184	lipoma HMGIC fusion partner-like 2
LOC388969	388969	hypothetical LOC388969
LRCH4	4034	leucine-rich repeats and calponin homology (CH) domain containing 4
LRRC8D	55144	leucine rich repeat containing 8 family, member D
LYPLA2	11313	lysophospholipase II
M6PR	4074	mannose-6-phosphate receptor (cation dependent)
MAP3K11	4296	mitogen-activated protein kinase kinase kinase 11

Gene Symbol	GeneID	Description
MAPK1	5594	mitogen-activated protein kinase 1
MARCKS	4082	myristoylated alanine-rich protein kinase C substrate
MARK2	2011	MAP/microtubule affinity-regulating kinase 2
MGC29891	126626	hypothetical protein MGC29891
MGC4268	83607	hypothetical protein MGC4268
MICAL3	57553	microtubule associated monoxygenase, calponin and LIM domain containing 3
MME	4311	membrane metallo-endopeptidase (neutral endopeptidase, enkephalinase, CALLA, CD10)
MOXD1	26002	monooxygenase, DBH-like 1
MRPS34	65993	mitochondrial ribosomal protein S34
MS4A6A	64231	membrane-spanning 4-domains, subfamily A, member 6A
MYO9B	4650	myosin IXB
NEK6	10783	NIMA (never in mitosis gene a)-related kinase 6
NIPA2	81614	non imprinted in Prader-Willi/Angelman syndrome 2
NRBF2	29982	nuclear receptor binding factor 2
NRIP3	56675	nuclear receptor interacting protein 3
OSCAR	126014	osteoclast-associated receptor
P2RX1	5023	purinergic receptor P2X, ligand-gated ion channel, 1
P2RX7	5027	purinergic receptor P2X, ligand-gated ion channel, 7
P2RY8	286530	purinergic receptor P2Y, G-protein coupled, 8
PABPC1	26986	poly(A) binding protein, cytoplasmic 1
PABPN1	8106	poly(A) binding protein, nuclear 1
PARP10	84875	poly (ADP-ribose) polymerase family, member 10
PDE4B	5142	phosphodiesterase 4B, cAMP-specific (phosphodiesterase E4 dunce homolog, Drosophila)
PFAS	5198	phosphoribosylformylglycinamidine synthase (FGAR amidotransferase)
PIP3-E	26034	phosphoinositide-binding protein PIP3-E
PLEK	5341	pleckstrin
PLK3	1263	polo-like kinase 3 (Drosophila)
POU2F2	5452	POU domain, class 2, transcription factor 2
PRDM1	639	PR domain containing 1, with ZNF domain
PRDM2	7799	PR domain containing 2, with ZNF domain
PREX1	57580	phosphatidylinositol 3,4,5-trisphosphate-dependent RAC exchanger 1
PRF1	5551	perforin 1 (pore forming protein)
PSD3	23362	pleckstrin and Sec7 domain containing 3
PTDSS1	9791	phosphatidylserine synthase 1
PTPN2	5771	protein tyrosine phosphatase, non-receptor type 2
PWWP2	170394	PWWP domain containing 2
RAB11FIP1	80223	RAB11 family interacting protein 1 (class I)
RAB11FIP4	84440	RAB11 family interacting protein 4 (class II)
RAPGEF6	51735	Rap guanine nucleotide exchange factor (GEF) 6
RARA	5914	retinoic acid receptor, alpha
RASSF2	9770	Ras association (RalGDS/AF-6) domain family 2
RASSF5	83593	Ras association (RalGDS/AF-6) domain family 5
RCE1	9986	RCE1 homolog, prenyl protein peptidase (S. cerevisiae)
RNF44	22838	ring finger protein 44
SAMD9	54809	sterile alpha motif domain containing 9
SAPS3	55291	SAPS domain family, member 3
SELL	6402	selectin L (lymphocyte adhesion molecule 1)
SEPHS2	22928	selenophosphate synthetase 2
SFRS2	6427	splicing factor, arginine/serine-rich 2
SFXN2	118980	sideroflexin 2
SFXN5	94097	sideroflexin 5
SGK	6446	serum/glucocorticoid regulated kinase
SGPL1	8879	sphingosine-1-phosphate lyase 1
SLA2	84174	Src-like-adaptor 2
SLC4A8	9498	solute carrier family 4, sodium bicarbonate cotransporter, member 8
SLC7A8	23428	solute carrier family 7 (cationic amino acid transporter, y+ system), member 8
SMARCD1	6602	SWI/SNF related, matrix associated, actin dependent regulator of chromatin, subfamily d, member 1
SMG5	23381	smg-5 homolog, nonsense mediated mRNA decay factor (C. elegans)
SNX30	401548	sorting nexin family member 30
SOCS1	8651	suppressor of cytokine signaling 1
SPRED1	161742	sprouty-related, EVH1 domain containing 1
SPTY2D1	144108	SPT2, Suppressor of Ty, domain containing 1 (S. cerevisiae)
SQLE	6713	squalene epoxidase
STXBP5	134957	syntaxin binding protein 5 (tomosyn)

Gene Symbol	GeneID	Description
SYAP1	94056	synapse associated protein 1, SAP47 homolog (Drosophila)
TBC1D9	23158	TBC1 domain family, member 9
TCF7	6932	transcription factor 7 (T-cell specific, HMG-box)
TFEC	22797	transcription factor EC
TGFBR1	7046	transforming growth factor, beta receptor I (activin A receptor type II-like kinase, 53kDa)
TGIF2	60436	TGFB-induced factor 2 (TALE family homeobox)
TGOLN2	10618	trans-golgi network protein 2
TMEM123	114908	transmembrane protein 123
TMEM127	55654	transmembrane protein 127
TMEM86A	144110	transmembrane protein 86A
TNFAIP3	7128	tumor necrosis factor, alpha-induced protein 3
TNFAIP8L2	79626	tumor necrosis factor, alpha-induced protein 8-like 2
TNFRSF10B	8795	tumor necrosis factor receptor superfamily, member 10b
TNFRSF19L	84957	tumor necrosis factor receptor superfamily, member 19-like
TNFRSF8	943	tumor necrosis factor receptor superfamily, member 8
TNK2	10188	tyrosine kinase, non-receptor, 2
TOMM40	10452	translocase of outer mitochondrial membrane 40 homolog (yeast)
TP53INP1	94241	tumor protein p53 inducible nuclear protein 1
TPM3	7170	tropomyosin 3
TRAF3	7187	TNF receptor-associated factor 3
UBE2J1	51465	ubiquitin-conjugating enzyme E2, J1 (UBC6 homolog, yeast)
VASH1	22846	vasohibin 1
VASP	7408	vasodilator-stimulated phosphoprotein
VHL	7428	von Hippel-Lindau tumor suppressor
VIL2	7430	villin 2 (ezrin)
WDR81	124997	WD repeat domain 81
WIPI2	26100	WD repeat domain, phosphoinositide interacting 2
YTHDF3	253943	YTH domain family, member 3
ZC3HAV1	56829	zinc finger CCCH-type, antiviral 1
ZNF217	7764	zinc finger protein 217
ZNF385	25946	zinc finger protein 385
ZNF652	22834	zinc finger protein 652

***Lenk GM, Tromp G, Weinsheimer S, Gatalica Z, Berguer R, and Kuivaniemi H.** Whole genome expression profiling reveals a significant role for immune function in human abdominal aortic aneurysms. *BMC Genomics* 8: 237, 2007.
